# The Effect of Orthographic Transparency on Auditory Word Recognition Across the Development of Reading Proficiency

**DOI:** 10.3389/fpsyg.2021.691989

**Published:** 2021-07-27

**Authors:** Mehdi Bakhtiar, Maryam Mokhlesin, Chotiga Pattamadilok, Stephen Politzer-Ahles, Caicai Zhang

**Affiliations:** ^1^Unit of Human Communication, Development, and Information Sciences, The University of Hong Kong, Hong Kong, China; ^2^Neuromuscular Rehabilitation Research Center, Semnan University of Medical Sciences, Semnan, Iran; ^3^Department of Speech Therapy, University of Social Welfare and Rehabilitation Sciences, Tehran, Iran; ^4^Aix Marseille University, CNRS, LPL, Aix-en-Provence, France; ^5^Department of Chinese and Bilingual Studies, The Hong Kong Polytechnic University, Hong Kong, China

**Keywords:** orthographic transparency, spoken word recognition, Persian script, reading acquisition, lexical decision

## Abstract

A question under debate in psycholinguistics is the nature of the relationship between spoken and written languages. Although it has been extensively shown that orthographic transparency, which varies across writing systems, strongly affects reading performance, its role in speech processing is much less investigated. The present study addressed this issue in Persian, whose writing system provides a possibility to assess the impact of orthographic transparency on spoken word recognition in young children at different stages of reading acquisition. In Persian, the long vowels are systematically present in the script, whereas the spelling correspondence of short vowels is progressively omitted from the script in the course of reading acquisition, thus, turning transparent into opaque spelling. Based on this unique characteristic, we tested 144 monolingual Persian-speaking nonreaders (i.e., preschoolers) and readers (second graders to fifth graders and young adults) in an auditory lexical decision task using transparent and opaque words. Overall, the results showed that, in accordance with the fact that the diacritics of short vowels are progressively omitted during the second year of schooling, the stimuli containing short vowels (opaque words) were recognized more slowly than transparent ones in third graders. Interestingly, there is a hint that the emergence of the transparency effect in the third graders was associated with an overall slower recognition speed in this group compared to their younger peers. These findings indicate that learning opaque spelling-sound correspondence might not only generate interference between the two language codes but also induce a general processing cost in the entire spoken language system.

## Introduction

A number of behavioral studies conducted on adults have provided evidence that orthographic knowledge has significant impacts on speech processing (Seidenberg and Tanenhaus, 1979; Dijkstra et al., 1995; Ziegler and Ferrand, 1998; Ziegler et al., 2003, 2004; Ventura et al., 2004; Peereman et al., 2009; Rastle et al., 2011; Pattamadilok et al., 2013; Qu and Damian, 2016; Qu et al., 2018). The first set of evidence came from metaphonological tasks, such as phoneme or rhyme monitoring. For instance, Seidenberg and Tanenhaus (1979) reported that making rhyming decisions was easier when spoken words shared both rhyme and rhyme spelling (e.g., pie-tie) than when the same spoken rhyme had different spelling (e.g., rye-tie). Following this initial observation, several research groups have investigated the influence of orthographic knowledge in more elementary spoken word recognition tasks, such as lexical decision or semantic decision. For instance, recognition of spoken words containing sounds with inconsistent sound-spelling mappings (those which can be spelled in different ways, such as /ip/ in *heap* vs. *deep*) is slower and more error-prone than recognition of spoken words composed of sounds with consistent sound-spelling mappings (such as the sound /oʊb/, in words like *probe* and *globe*; Ziegler and Ferrand, 1998; Ventura et al., 2004; Pattamadilok et al., 2007; Peereman et al., 2009). While most studies in the field have been investigating the impacts of orthography on participants’ native language and mainly in alphabetic writing systems, a growing number of studies reported similar observations in non-alphabetic languages, like Chinese, and in second language processing (Escudero et al., 2008; Veivo and Järvikivi, 2013; Qu and Damian, 2016; Qu et al., 2018). For instance, Tibetan-Chinese bilinguals were found to be slower in making semantic judgments when semantically unrelated spoken words were orthographically related compared to when words were both semantically and orthographically unrelated (Qu et al., 2018).

Regarding the mechanisms underlying the impacts of orthographic knowledge on speech processing, the current literature suggests that orthographic knowledge could affect speech processing through two complementary mechanisms (Muneaux and Ziegler, 2004; Pattamadilok et al., 2010). According to the *online co-activation account*, learning to read establishes connections between spoken and written codes of language such that seeing a word automatically activates its pronunciation and hearing a word automatically activates its spelling. Thus, in inconsistent or opaque writing systems, the mismatch between the orthographic and phonological representations would give rise to the competition between the two language codes and therefore interfere with word recognition (Grainger and Ferrand, 1996; Ziegler and Ferrand, 1998). This account is in line with the proposals of the connectionist models that assume a bidirectional connection between orthographic and phonological units at various levels of word processing (Stone et al., 1997; Harm and Seidenberg, 1999, but also see Norris et al., 2000): The presentation of spoken words is assumed to initially activate phonological units and then the corresponding orthographic units. This initial activation is followed by feedback from the orthographic units to the phonological ones. Thanks to the recurrent feedforward and feedback communication between the two language codes, a one-to-one correspondence between them facilitates speech recognition, while a mismatch between them hinders the recognition process (Stone et al., 1997; Ziegler and Ferrand, 1998; Muneaux and Ziegler, 2004).

However, the impacts of reading acquisition are not restricted to a simple connection between the two language codes. According to the *offline or developmental account*, the acquisition of a written code could alter the very nature of phonological representations throughout the learning process. As argued by Muneaux and Ziegler (2004), this mechanism could be compared to the lexical restructuring account claiming that phonological representations undergo important changes throughout language development (Metsala and Walley, 1998; Garlock et al., 2001). By introducing the orthographic code into the language system, this code could interfere with the existing language representations at several levels. For instance, it could lead to a reduction of the grain size of phonological representations (Goswami et al., 2005), a better specification of phoneme boundaries (Kolinsky et al., 2021), a modulation of the activation threshold of spoken words (Muneaux and Ziegler, 2004) or a transformation of phonological into ‘phonographic’ representations (Pattamadilok et al., 2014). Although these two mechanisms have mainly been examined in adult populations, one could reasonably assume that at the very beginning of reading acquisition when children learn to match sublexical speech units with the orthographic code, the online co-activation mechanism might play the most prominent role. The transformation of the nature of the phonological representations, probably at both sublexical and lexical levels as mentioned above, might occur later on and in a more progressive manner.

Much fewer studies have examined the effect of orthographic knowledge on speech processing in developmental populations. Some findings suggest that the extent to which speech processing performance in young children is affected by their orthographic knowledge also depends on the transparency of the writing system. Indeed, the regularity of sound-spelling correspondences varies considerably across alphabetic writing systems. For instance, sound-spelling correspondences are much more inconsistent (opaque) in French than in Portuguese. Testing Portuguese children, Ventura et al. (2007, 2008) observed significant effects of orthographic knowledge in third- to fourth-grade children in both lexical (lexical decision) and prelexical (shadowing) speech processing tasks. This observation suggested that, in a language that has a relatively transparent writing system like Portuguese, there is a strong connection between orthography and phonology at the sublexical level, which explained the generalized orthographic effect at both prelexical and lexical processing stages. Interestingly, a different pattern of result was reported when a similar experimental protocol was conducted in French: testing a population of second, third and fourth graders, Pattamadilok et al. (2009) replicated significant effects of orthography in both prelexical and lexical tasks previously reported in Portuguese only in the group of second graders. On the contrary, third and fourth graders showed a significant effect only in the lexical task, while no hint of a significant effect was found in the prelexical task. This restricted influence at the lexical stage was similar to that typically obtained in adult populations (Ziegler and Ferrand, 1998; Ventura et al., 2004; Pattamadilok et al., 2007). In other words, French beginning readers seemed to reach the adult pattern of the interaction between the phonological and orthographic system much earlier than young Portuguese readers. The authors explained the difference between the findings obtained in Portuguese and French by the difference in sound-spelling correspondences between the two writing systems: learning to read in an opaque writing system (like French) where the correspondence between phonemes and graphemes is irregular seems to push young readers (and probably school teachers) to abandon the pure sublexical decoding mechanism in favor of a lexical read-out earlier. This change of reading mechanism might as well have an impact on how orthographic knowledge influences speech processing. In the present case, it seemed to strengthen the connection between the spoken and written codes at the lexical stage and weaken their connection at the prelexical stage (Goswami et al., 2005; Ventura et al., 2007, 2008; Pattamadilok et al., 2009).

To our knowledge, so far, the comparison between opaque and transparent writing systems has been conducted across languages and, therefore, on children from different countries where different teaching methods might have been used. Thus, the discrepancies between different studies might not only be due to the writing systems, but also to other factors, such as the phonological system, or to the way the languages are formally taught. Furthermore, most of the previous studies that investigated the impact of orthographic transparency on spoken word recognition only considered the mismatch in sound-to-spelling direction. The present study proposed to fill this gap by examining the impact of orthographic transparency in spelling-to-sound direction on spoken word recognition within the same language where the same teaching method is applied to all children, and, more specifically, we investigated how this impact evolved with children’s education level. The main specificity of this study is the use of Persian with a unique feature of orthographic transparency in which some words that are considered orthographically transparent at the early stage of reading acquisition became orthographically opaque at the later educational stage (Baluch and Shahidi, 1991; Bakhtiar and Weekes, 2015; Rahbari, 2018). Persian is an Indo-European language written with an orthography adapted from that of Arabic, a Semitic language (Baluch, 2005). Persian has three long vowels which are obligatorily written as letter forms, as well as three short vowels which are not written in standard Persian. Thus, words which include only long vowels have transparent spelling-sound correspondences, whereas words with short vowels have opaque spelling-sound correspondences since they include sounds that are not represented in the written code. Furthermore, the (lack of) transparency of short vowels’ spelling follows a unique developmental trajectory: Persian short vowels can be optionally represented in writing using diacritics. These are used in early reading instruction for beginning readers (grades one and two). As learners progress to the higher grades (i.e., grades two and three), the diacritics are generally no longer written, and the children are exposed to the words in their non-vowelised opaque format. In other words, at the earliest stage of reading acquisition, all words are fairly transparent. However, as children reach grade two or three and start reading without diacritics, some formerly transparent words become opaque. Thus, a clear difference between transparent and opaque words may emerge around the transition from grade two to three. Studies of word naming (reading aloud) in Persian have shown that transparent words are indeed read faster and/or more accurately than opaque words by healthy children between the grades one to four (Baluch and Shahidi, 1991; Rahbari and Sénéchal, 2010), high school children (Rahbari and Sénéchal, 2008), healthy adults (Bakhtiar and Weekes, 2015) and people with aphasia (Bakhtiar et al., 2017). The relationship between orthographic transparency and reading procedure could be explained by the orthographic depth hypothesis (Katz and Frost, 1992). It has been argued that by increasing the orthographic depth, reliance on lexico-semantic route for word reading is increased (Schmalz et al., 2015). Previous studies in Persian (Bakhtiar and Weekes, 2015; Bakhtiar et al., 2017) have supported this assumption as reading the opaque words with stronger lexico-semantic features (e.g., highly imageable words) was faster than the opaque words with lower lexico-semantic features. Nevertheless the impact of orthographic depth has been well studied in visual word recognition; to our knowledge, the evidence on its impact on spoken word recognition is scarce, as this effect has mainly been examined in visual-based tasks.

To address this issue, we used an auditory lexical decision task to examine whether children’s auditory word recognition performance would be affected by orthographic transparency as they learned to read and write in different orthographic transparency formats, according to their education level. Our predictions are as follows. No difference in recognition performance would be observed between transparent and opaque words among preschoolers, who have not yet learned to read. A disadvantage for opaque words would emerge at grade two or three, as children learned to read in their non-vowelised format. The orthographic transparency effect might maintain at the later learning stages (grades 4 and 5) and even in adults, although the size of the effect might be reduced given that speech recognition would become increasingly fast and automatic for all word types in the latter groups of participants, especially for words that have early AoA (as used in the present study) or higher frequency (Seidenberg et al., 1984; Ziegler and Ferrand, 1998).

## Materials and Methods

### Participants

The participants were 144 right-handed healthy monolingual native Persian speakers (72 girls/women and 72 boys/men), including 24 preschool children (age range from 5.2 to 6.3, mean = 5.8, SD = 0.30) who were nonreaders, 24 s graders (age range from 7.3 to 8.3 mean = 7.8, SD = 0.34), 24 third graders (age range from 8.3 to 9.8 mean = 8.8, SD = 0.40), 24 fourth graders (age range from 9.3 to 10.4 mean = 10.8, SD = 0.35), 24 fifth graders (age range from 10.2 to 11.3 mean = 10.8, SD = 0.35) and 24 young adults (age range from 19.1 to 22.5 mean = 20.9, SD = 1.06) who were undergraduate students.^1^ Participants had no history of speech, language and hearing problems, and school-age children had no history of reading, writing or academic difficulties.^2^

All of the children passed the preschool auditory screening and IQ tests and their vocabulary skills were within the normal range based on the Persian version of picture vocabulary subtest of the Test of Language Development (Hassanzade and Minayi, 2009). Furthermore, one-way ANOVA showed a significant difference in vocabulary across the age groups [*F*(4.115) = 89.93, *p* < 0.001]. A Tukey post-hoc test revealed a normal developmental trend with an increase of vocabulary score with age: The higher age groups showed significantly better performance than the lower age groups (preschoolers: *M* = 22.25, SD = 1.07; grade two: *M* = 24.50, SD = 1.21; grade three: *M* = 25.70, SD = 1.23; grade four: *M* = 27.16, SD = 0.96; grade five: *M* = 27.83, SD = 1.23, all *p*s < 0.05, with the only exception that the difference between the grades 4 and 5 was not statistically significant, *p* = 0.26). This study was approved by the Ethics Committee of Semnan University of Medical Sciences, and informed consents were obtained from the participants or their caregivers.

### Stimuli

One hundred monosyllabic utterances were used, including 25 transparent words (/sib/, spelled *sib,* means ‘apple’), 25 opaque words (/sard/, spelled ‘*srd*’ means ‘cold’), 25 transparent pseudowords and 25 opaque pseudowords. The pseudowords were created based on the real words by changing one or two phonemes (e.g., /sos/, ‘sauce’ → /fos/). The words from the transparent and opaque conditions were closely matched based on the psycholinguistic norms developed for Persian monosyllabic words (Bakhtiar and Weekes, 2015), including the age of acquisition (AoA), frequency, imageability, neighborhood density, number of phonemes and acoustic duration. As discussed, the orthographic transparency in our study (unlike previous studies) refers to the presence/absence of mismatch in spelling-to-sound direction (rather than sound-to-spelling), which is respected to the absence of vowels in the print (see above examples). Therefore, in order to ensure that the mismatch between speech sounds and the written code only involved the vowels, the stimuli in the transparent and opaque conditions were matched on sound-to-spelling consistency of the consonants (i.e., the number of graphemes that can represent each consonant; see Table 1). Since the same stimuli were applied in all age groups, all words had relatively early AoA. Each stimulus was initially recorded three times by a female native speaker. The stimuli were annotated in Praat (Boersma and Weenink, 2019), and the most clearly produced token for each stimulus was selected. The mean acoustic intensity of all stimuli was normalized to 75 dB.

**Table 1:** Psycholinguistic variables for the opaque and transparent words (within each condition, means are shown on top and medians on the bottom).

	AoA	Freq	Img	Dens	N_Phon	SS_Con	AD
Opaque	3.1	17.6	6.6	11.3	3.4	0.9	647
	3.1	9.0	6.7	11.0	3.0	1.0	665
Transparent	3.0	17.6	6.7	10.6	3.4	0.9	658
	2.8	10.0	6.7	13.0	3.0	1.0	668

AoA, Age of acquisition; Freq, word frequency; Img, imageability; Dens, neighborhood density; N_Phon, number of phonemes; SS_Con, sound-spelling consistency and AD, acoustic duration.

### Procedure

The stimuli were presented to the participants by the DMDX software (Forster and Forster, 2003) in a quiet room. The subjects’ reaction times (RTs) measured at the onset of response production and response accuracy were recorded by DMDX. The stimuli were allocated into three blocks with closely equal number of words and pseudowords. Within each block, words and pseudowords were pseudorandomly intermixed and presented to the participants with a fixed order. No more than three opaque or transparent words/pseudowords were presented sequentially. Each trial began with a fixation cross presented in the centre of the screen for 500 ms. Then, an auditory stimulus was presented *via* headphones and participants were asked to judge whether it was a word or pseudoword as quickly and accurately as possible by pressing the left or right ALT buttons of the keyboard with their left or right index finger. The assignment of the left and right buttons to word vs. pseudoword responses was counterbalanced across participants. Eight practice trials with feedback were presented before the main test to familiarize the participants with the experimental procedure.^3^

### Data Analysis

The stimuli, data and analysis codes will be available at https://osf.io/y5tvx/. Four items (one opaque word and three transparent words) that were responded to incorrectly by over 50% of participants were removed from further analysis. Participants who responded incorrectly to over 50% of real-word trials were also removed from the analysis. These included seven preschoolers, 4 s graders, one third grader and one fifth grader. Trials with incorrect responses were removed from RT analysis, as were RTs longer or shorter than the mean ± 2.5 SD of each age group and each stimulus type. This led us to eliminate 2% of the remaining RT data.

We regressed RTs and accuracy on transparency (opaque vs. transparent), age group (preschoolers, grade two, grade three, grade four, grade five and university students) and their interaction. We coded the interaction as nested (group/transparency) in order to view the simple effect of transparency within each group. We also included random effects as described below. As discussed by Barr et al. (2013), it is ideal to include all random effects justified by the design. Therefore, random effects of group for participants are meaningless, since each participant was only a member of one group, and likewise random effects of transparency for items are meaningless for the same reason. Thus, the maximal random effects structure (including all random effects justified by the design), expressed in {lme4} syntax, would be (1 + Transparency|Participant) + (1 + Group|Item). This model also fits the correlation between the random participant intercepts and random effects of transparency for participants, and between the random item intercepts and random effects of group for items. As this model was too complex to fit without convergence errors, we then simplified the random effects structure following the guidelines suggested by Barr et al. (2013), specifically, preserving the random effect corresponding to the fixed effect of theoretical interest (i.e., transparency) while removing others if needed. Thus, the final model included only the random effect of transparency for participants and the random intercepts for items, and no random effect-intercept correlations; i.e. (0 + Transparency|Participant) + (1|Item). Statistical significance was evaluated using approximations of degrees of freedom as implemented in the lmerTest package in R (Luke, 2017).

## Results

**Figure 1:**
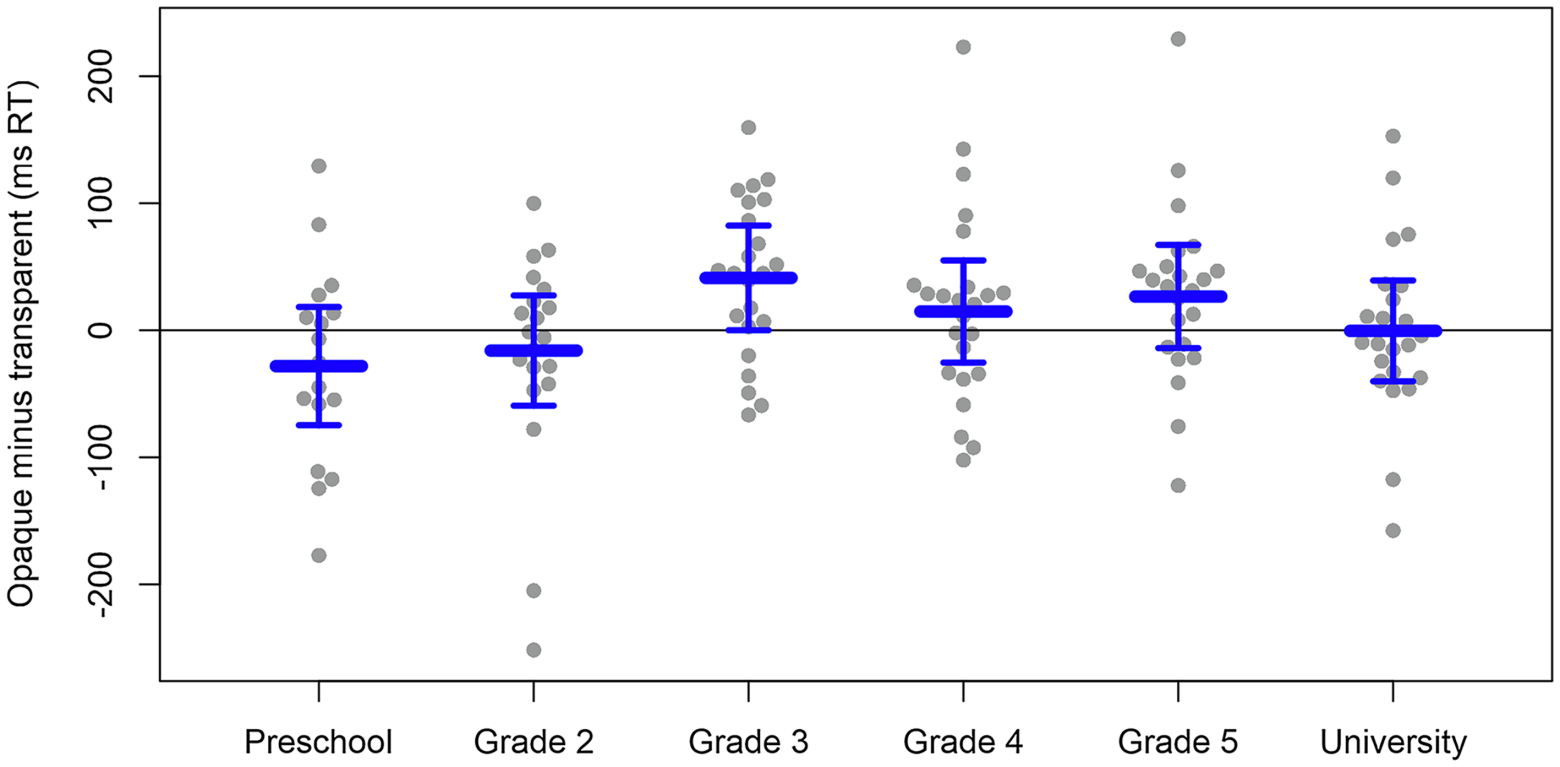
Means and 95% confidence intervals of the orthographic transparency effect (RT on opaque stimuli minus RT on transparent stimuli) obtained of each age group. The dots represent the individual data.

Table 2 reports the mean and standard errors for the transparent and opaque stimuli across different age groups. Figure 1 shows the size of transparency effects (RT for opaque words minus RT for transparent words) for each participant, arranged by age group, along with the 95% confidence interval of the transparency effect in each age group from the mixed-effects model. As suggested by Figure 1, only third graders appear to show a significant transparency effect (within the third graders, most participants’ transparency effect is around 50 milliseconds, and the 95% confidence interval does not include zero). For the other age groups, the transparency effect clustered near zero and the 95% confidence intervals, including zero. Statistical analysis confirmed these impressions.

**Table 2:** Mean ± SE for each group and condition.

	Opaque words	Transparent words	Opaque pseudowords	Transparent pseudowords
Preschool	1,449 ± 63 (33)	1,478 ± 73 (35)	1,566 ± 83 (27)	1,597 ± 80 (23)
Grade 2	1,376 ± 48 (22)	1,409 ± 49 (27)	1,497 ± 48 (21)	1,503 ± 54 (23)
Grade 3	1,514 ± 44 (21)	1,455 ± 48 (26)	1,625 ± 54 (12)	1,589 ± 59 (16)
Grade 4	1,417 ± 46 (16)	1,408 ± 52 (20)	1,526 ± 46 (8)	1,503 ± 41 (10)
Grade 5	1,412 ± 56 (14)	1,397 ± 60 (18)	1,539 ± 69 (7)	1,486 ± 57 (9)
University	1,258 ± 27 (4)	1,246 ± 26 (10)	1,338 ± 27 (4)	1,342 ± 28 (5)

Error percentage is given in parentheses.

The interaction between transparency and age group was significant, as indicated by a significantly better fit for a model, including this interaction compared to a maximally similar model without the interaction [*χ*^2^(5) = 12.68, *p* = 0.027]. Since the examination of the transparency effect within each age group is the aim of the study, we conducted planned comparisons on the RTs obtained in the transparent and the opaque conditions within each group. As suggested by Figure 1, there is a significant transparency effect in third graders (*t* = −1.98, *p* = 0.049), with transparent words being processed 41 ms faster than opaque words. Grades 4 and 5, as shown in Figure 1, showed numerical effects in this direction, but these were not significant (*p* = 0.469 and *p* = 0.198, respectively). Although preschoolers and second graders showed numerical effects in the opposite direction (i.e., slower for transparent than for opaque), these differences were not statistically significant (*p* = 0.233 and *p* = 0.467, respectively).

We also examined lexical decision accuracy results in which accuracy increased as a function of age group: every age group responded significantly more accurately than the previous age group, as indicated by a logistic mixed effects model with forward difference contrast coding and the same random effects structure as the analysis described above (*p*s < 0.001). Regarding the transparency effect, the only difference between the two stimuli types was found among university students (*b* = 0.67, *z* = 2.61, *p* = 0.009). Unexpectedly, the result reflects an advantage of opaque over transparent stimuli.

**Figure 2:**
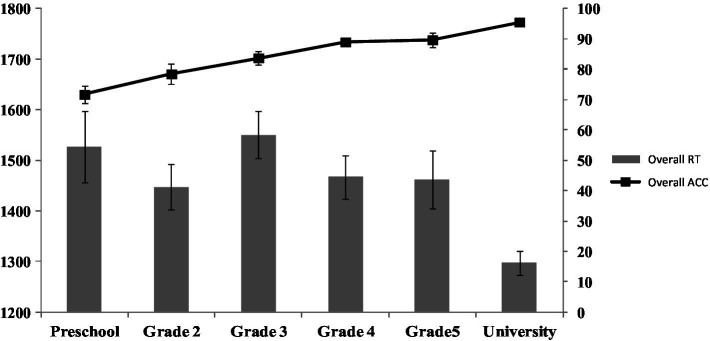
Overall speech recognition performance obtained on all stimulus types. The left y-axis (bar plots) represents the RTs, and the right y-axis (line plots) represents the accuracy scores obtained in the different groups of participants. Error bars represent the standard errors of the mean.

Lastly, we conducted an exploratory analysis to explore the evolution of the overall speech recognition performance regardless of transparency. As discussed, the overall accuracy increased as a function of age group as the general language abilities of healthy individuals are expected to increase with age. However, inspection of the RT data (shown in Figure 2) revealed an interestingly different result pattern, which was also confirmed in exploratory mixed-effects models. While the university students’ reaction times were, as expected, faster than all other groups’ (all *p*s < 0.05), the mean RT obtained in third graders, i.e., those who showed a significant orthographic transparency effect in the former analysis, was somewhat longer that the one observed in the group of second graders (*p* = 0.06) and was not significantly different than that of preschoolers (see Figure 2). It should be noted that these results are exploratory so they should be taken with a grain of salt.

## Discussion

Based on the specificity of Persian’s writing system where orthographic transparency is progressively reduced for children, we conducted a behavioral study to test the assumption that acquiring a written code has an impact on one’s ability to process speech and that the impact might vary with participants’ education level. Using an auditory lexical decision task in which performances on stimuli with transparent and opaque orthography were compared, we obtained evidence in line with our assumption: while no significant difference between stimuli with transparent and opaque orthography was found on preschoolers, stimuli with transparent orthography were recognized faster than those with opaque orthography in third graders, that is, the moment when the spelling markers of short vowels are completely removed from the script. Before that age, these vowels were either fully presented (first grade) or partially presented (second grade) in the written script as diacritics for learning the new words, which made their spellings remain relatively transparent. This early phase of the transition from transparent to opaque spelling may explain the absence of the effect in second graders. According to the online co-activation account, it can be speculated that the complete transition from transparent to opaque spelling in third graders (unlike the second graders) aborts the consistent facilitating feedback from the orthographic units (diacritics) to the phonological units (short vowels), which results in slower processing of the opaque words (than transparent words) in this age group compared to second graders. It is also notable that this research would support the previous findings that reported the significant effects of orthographic knowledge on auditory lexical decision among third graders in French (Pattamadilok et al., 2009) and Portuguese (Ventura et al., 2007, 2008). However, unlike those studies, we cannot tease apart the effects of orthographic knowledge at the sublexical versus lexical levels. Further study may look into this effect by using different tasks that may tap into the lexical and sublexical word processing in Persian.

However, the absence of the transparency effect in the older age groups was unexpected and required further explanations. One reason would be that the stimuli used in the present studies were designed for young children as they had relatively early AoA. Our previous research conducted on adults has shown that the early acquired opaque words led to the same recognition performance as early acquired transparent words in visual lexical decision task in Persian (Bakhtiar et al., 2016). Therefore, it could be assumed that the impact of a mismatch between the spoken and written code at the sublexical level would be negligible at the older ages or when the frequency of exposure is increased. As previously discussed by Ziegler and Ferrand (1998); see also Seidenberg et al. (1984), a greater amount of learning for high frequency words would allow a rapid word recognition at a whole word level. As a result, the impact of any manipulation that occurs at a smaller grain size might be reduced or disappear. Moreover, the absence of the transparency effect in the older age groups is coherent with existing evidence. For instance, a recent study conducted on Persian-speaking children (Rahbari, 2018) reported that the opaque words were spelled as comparably accurate as the transparent words in a dictation task, which require auditory word recognition, but less accurately in reading task. This may confirm the assumption that the effect size of orthographic transparency is smaller in auditory word recognition than visual word recognition (Ziegler et al., 2008). However, as we also tested the RT responses (unlike Rahbari, 2018), we found that the effect of orthographic transparency can be traced in auditory word recognition processing as well (albeit to a lesser extent). An alternative hypothesis would be that since the major shift from fully transparent (with diacritics) to opaque (without diacritics) writing system happens at the third grade, the effect of orthographic transparency is more robust in this age group, whereas it could be negligible in older age groups and in adults who are more familiar with the absence of diacritics for short vowels in the print. However, although our young children were recruited based on their good level of spelling knowledge (as reported by their teachers), a stricter control of spelling knowledge on the items used in the study would allow us to ascertain that the reduced or absence of orthographic knowledge was indeed due to children’s familiarity with the absence of diacritics for short vowels rather than to the possibility that some children might not know the spelling of the critical words well enough.

One intriguing finding is that the adult group recognized spoken words with opaque orthographies more accurately than spoken words with transparent ones. Although this pattern is difficult to explain and require further research, one explanation can be proposed in the light of previous research on word recall in Persian (Baluch and Danaye-Tousi, 2006a). Baluch and Danaye-Tousi (2006a) reported that the older/more skilled readers were able to recall the orthographically opaque words more accurately than the transparent words (but also see Baluch and Danaye-Tousie, 2006b, for different results), whereas the younger/beginning readers and dyslexic groups showed an opposite pattern. The advantage of word recall for opaque words among skilled readers was argued in relation to the ‘depth of processing’ at the encoding time, which might be greater for opaque words as they are more relying on lexico-semantic processes than transparent words. Another speculation could be related to the distribution of opaque versus transparent words in Persian script for adults. Our previous research has used a metric to calculate the degree of orthographic transparency (DT) by dividing the number of letters by the number of phonemes (Bakhtiar and Weekes, 2015). It was found that the completely transparent words (DT = 1) only comprise 11% of the word units in a Persian corpus for adults, whereas 89% of them were partially opaque words with DT ranged between 0.50 and 0.92 (Bakhtiar and Weekes, 2015). This indicates that with language experiences, the adult readers may generally become more familiar with opaque rimes despite the fact that the objective frequency values of the opaque and the transparent words used in the present study were matched. This could explain the unexpected better performance on opaque orthography than transparent one observed here.

A final finding that deserves further attention is the observation that the emergence of the transparency effect in the third graders coincided with an overall slowdown of recognition speed in this group compared to younger, second grade, children. Generally, one could expect an improvement of language abilities across ages, as reflects in an overall increase of accuracy scores and a reduction of processing speed. Although this typical developmental pattern was found on the accuracy scores, the RT data remained puzzling. One possible explanation is that being exposed to the opaque form of the written words, which is the most natural format of the script not only generates interference between the two language codes but also induces a general instability and thus increases processing cost in the entire spoken language system. This interpretation is supported by the existing literature showing that learning to read also induces profound changes within the spoken language system that are far beyond a simple connection between the two language codes (Dehaene et al., 2010; Brennan et al., 2013). As was previously suggested by Muneaux and Ziegler (2004), that is, acquiring an orthographic code could further contribute to the on-going lexical restructuring process. This account initially claims that phonological representations undergo important changes throughout the language development (Metsala and Walley, 1998; Garlock et al., 2001). According to the offline or developmental account that was discussed earlier, learning to read could modify the organization as well as the nature of the existing phonological representations. We argued that these changes would be particularly destabilizing for the still-developing spoken language system in young children and thus probably lead to an overall (although transient) slowdown of spoken word recognition as reported here. To confirm the causal relationship between learning to read in an opaque writing system and a general slowdown of the spoken language system reported here, one should investigate this phenomenon in a larger sample size using wide-range measures of spoken and written language abilities, and ideally by applying a longitudinal protocol.

## Data Availability Statement

The datasets presented in this study can be found in online repositories. The names of the repository/repositories and accession number(s) can be found in the article/supplementary material.

## Ethics Statement

The studies involving human participants were reviewed and approved by The ethics committee of Semnan University of Medical Sciences (IR.SEMUMS.REC.1397.217). Written informed consent to participate in this study was provided by the participants’ legal guardian/next of kin.

## Author Contributions

MB involved in developing the original research plan, stimuli and task preparation, writing the original draft, and revision of the manuscript. MM involved in supervision of data collection, stimuli and task preparation, and grant preparation. CP involved in developing the original research plan, task design, and revision of the manuscript. SP-A involved in running the statistical analysis and drafting the relevant parts in the manuscript. CZ, involved in developing the original research plan, writing and revision of the manuscript. All authors contributed to the article and approved the submitted version.

## Conflict of Interest

The authors declare that the research was conducted in the absence of any commercial or financial relationships that could be construed as a potential conflict of interest.

## Publisher’s Note

All claims expressed in this article are solely those of the authors and do not necessarily represent those of their affiliated organizations, or those of the publisher, the editors and the reviewers. Any product that may be evaluated in this article, or claim that may be made by its manufacturer, is not guaranteed or endorsed by the publisher.

## Footnotes

^1^Since data collection was conducted in the middle of the education year, it was difficult to know whether and to what extent the children in the first grade had received reading and writing instruction. Therefore, we chose to include in the study only participants who were clearly pre-readers (i.e., preschoolers) or readers.

^2^We did not specifically test the spelling knowledge of the word stimuli in the experiment among different grades. However, reader participants were recruited based on their dictation performance reported by their teachers. Only children with moderate to excellent dictation scores who also learned to reasonably eliminate the short vowels in their dictation task were included in the study.

^3^We also ran a shadowing task about 2 weeks after the lexical decision task, which is not reported here since multiple participants did not return for the shadowing task, and also due to the uncertainty over the effects of participants’ familiarity with stimuli on the final results.
